# Outcome after surgery for metastatic spinal cord compression in 54 patients with prostate cancer

**DOI:** 10.3109/17453674.2011.590761

**Published:** 2012-02-08

**Authors:** Sead Crnalic, Christer Hildingsson, Pernilla Wikström, Anders Bergh, Richard Löfvenberg, Anders Widmark

**Affiliations:** ^1^Departments of Surgical and Perioperative Sciences (Orthopedics); ^2^Medical Biosciences (Pathology); ^3^Radiation Sciences (Oncology), Umeå University, Umeå, Sweden

## Abstract

**Background and purpose:**

The criteria for selecting patients who may benefit from surgery of spinal cord compression in metastatic prostate cancer are poorly defined. We therefore studied patients operated for metastatic spinal cord compression in order to evaluate outcome of surgery and to find predictors of survival.

**Patients and methods:**

We reviewed the records of 54 consecutive patients with metastatic prostate cancer who were operated for spinal cord compression at Umeå University Hospital. The indication for surgery was neurological deficit due to spinal cord compression. 41 patients had hormone-refractory cancer and 13 patients had previously untreated, hormone-naïve prostate cancer. 29 patients were operated with posterior decompression only, and in 25 patients posterior decompression and stabilization was performed.

**Results:**

Preoperatively, 6/54 of patients were able to walk. 1 month after surgery, 33 patients were walking, 15 were non-ambulatory, and 6 had died. Mortality rate was 11% at 1 month, 41% at 6 months, and 59% at 1 year. In the hormone-naïve group, 8/13 patients were still alive with a median postoperative follow-up of 26 months. In the hormone-refractory group, median survival was 5 months. Patients with hormone-refractory disease and Karnofsky performance status (KPS) of ≤ 60% had median survival of 2.5 months, whereas those with KPS of 70% and KPS of ≥ 80% had a median survival of 7 months and 18 months, respectively (p < 0.001). Visceral metastases were present in 12/41 patients with hormone-refractory tumor at the time of spinal surgery, and their median survival was 4 months—as compared to 10 months in patients without visceral metastases (p = 0.003). Complications within 30 days of surgery occurred in 19/54 patients.

**Interpretation:**

Our results indicate that patients with hormone-naive disease, and those with hormone-refractory disease with good performance status and lacking visceral metastases, may be helped by surgery for metastatic spinal cord compression.

Bone metastasis occurs in more than 80% of patients with advanced prostate cancer, most commonly in the spine (Bubendorf et al. 2000). Spinal cord compression usually occurs in patients with advanced hormone-resistant disease, causing neurological complications detrimental to quality and duration of life (Clarke 2006). However, spinal cord compression may also be the first clinical manifestation of metastases in patients with previously localized disease, or may occasionally be the presenting sign in patients with previously unrecognized prostate cancer. Spinal cord compression in patients with prostate cancer has been reported to have an incidence of 3–7% (Kuban et al. 1986, Honnens de Lichtenberg et al. 1992, Loblaw et al. 2003).

The outcome of surgery for spinal cord compression is usually reported in series involving different tumors, making it difficult to draw conclusions on specific tumor types (Jansson and Bauer 2006, Chaichana et al. 2009). In some studies limited to prostate cancer, surgical treatment has been analyzed together with results of radiation treatment, with a low number of patients operated (Huddart et al. 1997, Cereceda et al. 2003, Tazi et al. 2003). There have only been a few studies that have specifically addressed surgical treatment of metastatic spinal cord compression in prostate cancer (Shoskes and Perrin 1989, Williams et al. 2009). Furthermore, the criteria for selecting patients who may benefit from surgical therapy of spinal cord compression are poorly defined.

We therefore studied patients with prostate cancer who were operated for metastatic spinal cord compression to evaluate outcome of surgery and to find predictors of survival and neurological restitution.

## Patients and methods

We reviewed the records of 54 consecutive patients with histologically proven prostate cancer who were operated for metastatic spinal cord compression at Umeå University Hospital, Sweden, between September 2003 and December 2008. The material for morphological studies was collected prospectively as part of a larger study on bone metastasis from prostate cancer, and 53 patients from the present study were included in the study on androgen receptor expression in bone metastases (Crnalic et al. 2010). The study was approved by the local ethics review board (No. 223/03, dnr 03-185 and dnr 04-26M).

The indication for surgery was neurological deficit due to spinal cord compression. 41 patients had already been diagnosed as having hormone-refractory prostate cancer, whereas 13 patients had previously untreated, hormone-naïve prostate cancer (Table 1). The surgical and medical therapies were not randomized, and were performed according to the preferences of the surgical and oncology teams. A high dose of steroids was prescribed to 51 of the 54 patients after the onset of neurological symptoms.

**Table 1.** Clinical characteristics of 54 patients with prostate cancer who were operated for metastatic spinal cord compression (SCC)^a^

		Hormone status	
		Hormone-naïve **^b^**	Hormone-refractory **^c^**
Clinical characteristics		(n = 13)	(n = 41)
Age at diagnosis of primary tumor		77 (60–85)	69 (51–86)
Age at surgery for SCC		77 (60–85)	72 (54–88)
PSA at diagnosis of primary tumor (ng/mL)		140 (21–4,000)	82 (2–7,300)
PSA at surgery for SCC (ng/mL)		140 (21–3,704)	190 (0.5–5,139) **^d^**
Interval between primary tumor diagnosis and surgery for SCC (months)		0	34 (3.5–216)
Gleason score of primary tumor:	6	0	2
	7	1	14
	8	1	6
	9	1	6
	10	0	3
	Not available	10	10
Other sites of metastasis at the time of surgery for SCC **^e^**			
Abdominal organs	0	3
Pelvic organs		0	3
Lymph nodes		0	9
Lung		0	3
Other bones		11	41
Preoperative Frankel grade: **^f^**	A	0	0
	B	0	3
	C	12	33
	D	1	5
	E	0	0

**^a^** Values are given as median (min–max) or absolute numbers (number of patients).

**^b^** Diagnosed with prostate cancer as a result of pain or neurological symptoms from spinal metastasis.

**^c^** Patients with disease progression after long-term androgen deprivation therapy.

**^d^** PSA values were not available for 3 patients.

**^e^**Visceral metastases were registered in 12 patients in the hormone-refractory group. Patients may have more than one metastasis.

**^f^**Grade A: complete lesion (paraplegia); grade B: only sensory function; grade C: motor function present but not of practical use (non-ambulatory); grade D: motor function present, sufficient to allow walking (ambulatory); grade E: no neurological symptoms.

Treatment of primary prostate cancer for the patients in the hormone-refractory group consisted of androgen deprivation therapy, either with luteinizing hormone-releasing hormone (LHRH) agonists (33 patients) or orchiectomy (8 patients). 1 patient underwent radical prostatectomy, and 3 patients received curative radiation therapy (78 Gy). In addition, 29 patients also received antiandrogens. After failure of hormone treatment, 5 patients were given chemotherapy. Skeletal and non-skeletal metastases were treated with palliative radiotherapy (17 patients). 5 of these patients received palliative radiotherapy because of continuous pain, to the same spinal level as the operation site, at a median interval of 5 (4–18) months before spinal surgery. 1 patient had radiation treatment due to neurological symptoms 9 days before surgery for spinal cord compression. 3 patients received bisphosphonates (zolendronic acid) and 4 were given radioisotopes. In addition, 11 patients were on continuous therapy with low-dose prednisone, mainly for pain relief, for median 6 (1.5–12) months until the time of surgery.

The 13 patients with hormone-naïve prostate cancer were treated with androgen ablation (orhiectomy 12 patients, LHRH agonist 1 patient) either a short time (2–7 days) before spinal surgery (4 patients) or immediately afterwards (9 patients).

The anatomic location of spinal lesions was assessed by preoperative MR imaging (Table 2). Neurological function was graded according to Frankel preoperatively, and 4 weeks and 6 months postoperatively. The preoperative functional status of the patients before presentation with neurological symptoms had been evaluated according to the Karnofsky performance status scale (KPS). The follow-up time was defined as the time between the date of operation and the latest follow-up examination or death. The median follow-up time for survivors was 26 (7–52) months in the hormone-naïve group and 12 (7–27) months in the hormone-refractory group.

**Table 2.** Anatomic localization of spinal cord compression^a^

Anatomical site	Anatomical distribution			
	1 level	2 levels	3 levels	4 levels
Cervical		1		
Thoracic	28	14 **^b^**	1	1 **^c^**
Lumbar	7	2		

**^a^** Data represent number of patients.

**^b^** In two patients, 2 separate levels were involved: Th4 + Th8, and Th6 + Th9, respectively.

**^c^** Levels involved: C7-Th3.

### Statistics

Groups were compared with the Mann-Whitney U-test. Postoperative survival was estimated by Kaplan-Meier analysis with death from prostate cancer as event. Survival curves were compared with the log-rank test. A p-value of ≤ 0.05 was considered statistically significant. Statistical analysis was performed using GraphPad Prism software version 5.0 (GraphPad Inc., San Diego, CA).

## Results

### Surgical procedure and postoperative treatment

29 patients were operated with posterior decompression, and 25 patients with posterior decompression and stabilization with pedicle screws or with pedicle screws and hooks. The median operating time was 1.3 (0.6–3.2) h for posterior decompression and 2.7 (1.3–5.9) h for posterior decompression and stabilization. Adjuvant radiation therapy was given to 35 patients (11 hormone-naïve, 24 hormone-refractory) at a median dose of 28 (16–28) Gy and at median interval of 33 (18–69) days postoperatively. 10 patients (1 hormone-naïve, 9 hormone-refractory) received chemotherapy and 8 patients (1 hormone-naïve, 7 hormone-refractory) received bisphosphonates postoperatively.

### Neurological function postoperatively

33 of the 54 patients were able to walk 1 month after surgery (Table 3). 27 of 45 patients in the preoperative Frankel grade C group regained their ability to walk 4 weeks after surgery; 9 of them had hormone-naïve tumors and 18 had hormone-refractory tumors. 1 patient with hormone-naïve tumor was operated for subdural hematoma 1 week before spinal surgery, and 1 patient with hormone-refractory tumor suffered a stroke 3 weeks postoperatively; none of them improved in neurological function (Frankel grade C) postoperatively. All 6 patients with Frankel grade D preoperatively retained their neurological function 4 weeks postoperatively. None of the patients from the Frankel grade B group reached ambulatory status.

**Table 3.** Neurological evaluation using the Frankel^a^ classification: preoperative values compared with those recorded 4 weeks postoperatively

	Pre-op.	4 weeks post-op.					
	n=54 **^b^**	A	B	C	D	E	Dead
Hormone-refractory							
A							
B	3 **^b^**			2			1
C	33 **^b^**			10	18		5
D	5				5		
E							
Hormone-naive							
A							
B							
C	12			3	9		
D	1				1		
E							

**^a^**See legends to Table 1.

**^b^** 6 patients with hormone refractory tumors died within 4 weeks after operation and are not included in 4 weeks postoperative evaluation.

None of the 15 patients with grade C at 4 weeks postoperatively improved neurologically on later follow-up. Of the 33 patients who were ambulatory at 4 weeks, 27 were still alive at 6 months and 26 of them had retained their neurological function, whereas 1 patient could not walk. 24 of 35 patients with 1 level of spinal cord compression and 9 of 19 patients with ≥ 2 levels of spinal cord compression were ambulatory at 4 weeks. 16 of 29 patients who were operated with posterior decompression and 17 of 25 patients who were operated with posterior decompression and stabilization were ambulatory 4 weeks after surgery.

### Complications

24 complications were recorded within 30 days of surgery in 19 of the 54 patients (Table 4). Systemic complications occurred in 9 patients, local complications in 8, and 2 patients had both local and systemic complications. 5 of the 11 patients with systemic complications and 2 of the 10 patients with local complications also had visceral metastases at the time of surgery. 1 patient with wound dehiscence had received radiation therapy to the operation site 9 days preoperatively. 11 patients who were operated with posterior decompression had complications (5 systemic, 8 local) as compared to 8 patients who were operated with posterior decompression and stabilization (6 systemic, 5 local). In the hormone-naïve group, 2 patients had complications (2 systemic, 2 local). In the hormone-refractory group, 17 patients had complications (9 systemic, 11 local). The postoperative complication rate was not related to age (p = 0.7).

**Table 4.** Complications of surgery for metastatic spinal cord compression that were registered in 19 of 54 patients within 30 days of surgery (patients may have more than one complication)

Systemic complications	n **^a^**	n **^b^**	Follow-up after surgery	
Thromboembolic: stroke		1	3 months died	
Pulmonary: pneumonia	1	2	1 **^b^** and 5 **^a^** months died; and 14 **^b^** months alive	
embolism		1	7 months alive	
Septicemia	1		12 days died	
Gastrointestinal:				
intestinal rupture	1		10 days died	
bleeding		1	12 days died	
Multiple organ failure	2	1	5 **^a^**, 16 **^b^**, and 28 **^a^** days died	
**Local complications**	**n ^a^**	**n ^b^**	**Revised ^c^**	**Follow-up after surgery**
Superficial wound infection	3	2	1 **^b^**	2 **^a^**, 5 **^a^**, 7 **^b^**, and 16 **^a^** months died; and 14 **^b^** months alive
CSF leak	1	1	1 **^a,d^**	4 **^a^** months died; and 14 **^b^** months alive
Wound dehiscence	1	1	2	4 **^a^** and 7 **^b^** months died
Sacral pressure sores	3	1	2 **^a^**	1 **^a,e^**, 1.5 **^b^**, 2 **^a^**, and 10 **^a^** months died

**^a^** Posterior decompression.

**^b^** Posterior decompression and stabilization.

**^c^** In all cases, local surgical wound revision was performed, and in the patient with CSF leakage the dural rift was sutured and the wound was covered with musculocutaneous flap.

**^d^** Revised twice.

**^e^** Patient was also operated for pathological fracture of the left femur 10 days after spinal surgery.

### Survival

**Figure 1.**
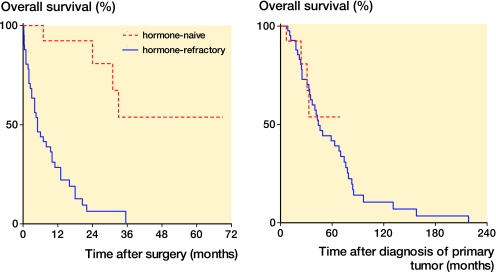
Survival for the patients with hormone-naïve (n = 13) and hormone-refractory (n = 41) prostate cancer after surgery for spinal cord compression (left), and after diagnosis of the primary tumor (right).

**Figure 2.**
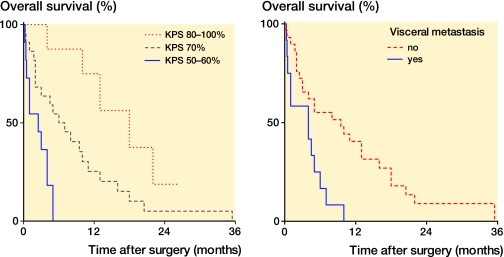
Survival for the patients with hormone-refractory prostate cancer (n = 41) according to Karnofsky performance status **^a,b^** (KPS) (left), and presence of visceral metastasis (right).

**Figure 3.**
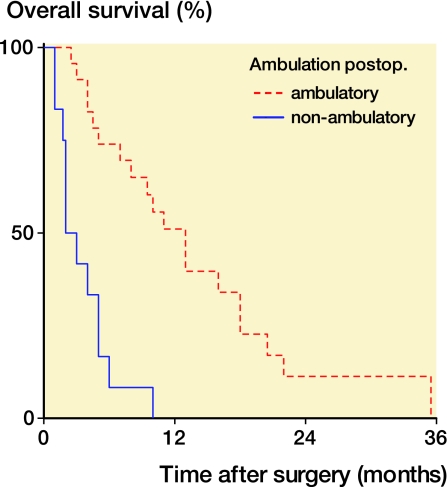
Survival for the patients with hormone-refractory prostate cancer (n = 35) **^a^** according to ambulatory status 4 weeks after surgery for spinal cord compression.

6 patients died within 30 days of surgery. Mortality rate was 11% at 1 month, 24% at 3 months, 41% at 6 months, and 59% at 1 year. At the latest follow-up, 8 patients in the hormone-naïve group and 4 patients in the hormone-refractory group were still alive after median postoperative follow-up times of 26 (7–52) months and 12 (7–27) months, respectively. In the hormone-naïve group, more than half of the patients were still alive at the latest follow-up, whereas there was a postoperative median survival of only 5 (0.25–36) months in the hormone-refractory group (p < 0.001) (Figure 1a). Patients with hormone-refractory disease and bad performance status (KPS ≤ 60%, n = 11) had a median survival of 2.5 (0.25–5) months, whereas those with a KPS of 70% (n = 22) and a KPS of ≥ 80% (n = 8) had median survival of 7 (0.3–36) and 18 (4–27) months, respectively (p < 0.001) (Figure 2a). In the group with KPS ≤ 60%, 5 patients had visceral metastases at the time of surgery and 7 patients suffered from complications within one month of surgery. 12 of 41 patients with hormone-refractory tumor had visceral metastases at the time of spinal surgery. Their median survival was 4 (0.3–10) months, as compared to 10 (0.25–36) months for patients without visceral metastases (p = 0.003) (Figure 2b). Patients with hormone-refractory tumor who were ambulatory 4 weeks after surgery generally had longer survival times than non-ambulatory patients, with median survival of 13 (2.5–36) and 2.5 (1–10) months, respectively (p < 0.001) (Figure 3). Survival was not found to be related to age when ages of 65, 70, or 75 years were used as cut-off in survival analysis—either in the whole patient material (p = 0.7, p = 0.1, and p = 0.2) or in the hormone-refractory group analyzed separately (p = 0.5, p = 0.9 and p = 0.8). There was no statistically significant difference in the survival of patients when the two surgical methods were compared (p = 0.6).

**^a^** 100: normal, no complaints, no evidence of disease; 90: able to carry on normal activity, minor signs of symptoms of disease; 80: normal activity with effort, some signs or symptoms of disease; 70: cares for self, unable to carry on normal activity or to do any work; 60: requires occasional assistance from others but able to care for most of own needs; 50: requires considerable assistance from others and frequent medical care; 40: disabled, requires special care and assistance; 30: severely disabled, hospitalization indicated, death not imminent; 20: very sick, hospitalization necessary, active supportive treatment necessary; 10: moribund; 0: dead.
**^b^** No patients had KPS less than 50%.

**^a^** 6 of 41 patients died within 4 weeks of surgery and were not included in the survival analysis related to ambulatory status.

## Discussion

We found that the hormone status of bone metastases, the preoperative performance status of the patient, and preoperative morbidity related to the presence of visceral metastases had an influence on outcome after surgery for spinal cord compression in prostate cancer. Our results also indicate that surgery may improve or preserve neurological function in patients with metastatic spinal cord compression.

The limitations of the present study are the small number of patients, the fact that the surgical or medical therapy was not randomized, and its retrospective character (although it was part of a larger prospectively collected material). The strength of the study is that it describes surgical outcome for a specific tumor type in a consecutive series of patients with the same indication for surgery.

Only one tenth of the patients in our study were able to walk preoperatively. 1 month after surgery, more than half of them were walking. Neurological deficit was the only inclusion criterion in this study, whereas intractable pain with or without deformity or impaired neurological function were inclusion criteria in the other 2 studies on surgical treatment of spinal cord compression in prostate cancer. Shoskes and Perrin 1989) reported on 28 patients and Williams et al. 2009) reported on 44 patients; both studies included patients with previously treated prostate cancer. Unlike our patients, 32 of 44 (Williams et al. 2009) and 15 of 28 (Shoskes and Perrin 1989) were ambulatory preoperatively. 8 of 12 patients in the study by Williams et al. 2009) and 8 of 13 patients in the study by Shoskes and Perrin 1989) who were non-ambulatory preoperatively regained their ability to walk postoperatively, rates which are similar to the 27/48 patients in our study. Median survival in patients with hormone-refractory tumors in our study was 5 months—the same as in the series of Williams et al. 2009), suggesting similar disease burden. Our patients are comparable to those of Jansson and Bauer 2006), who reported on surgical outcome of spinal cord compression in 282 patients with different tumors. Two-thirds of their patients could not walk before surgery and 23% had visceral metastases. 40% of the patients had prostate cancer with results on survival similar to our results. The complication and mortality rates in our study are similar to those reported in a population-based study of surgery for spinal metastases in 987 patients, 137 of whom had prostate cancer (Finkelstein et al. 2003).

Prolonged functional independence may potentially improve quality of life and survival in carefully selected patients. Thus, the results of our study—as in other retrospective and prospective observational studies—demonstrate a longer median survival time in patients who are able to walk after surgery or radiotherapy than in non-ambulatory patients (Hill et al. 1993, Hirabayashi et al. 2003, Helweg-Larsen et al. 2000).

Regarding preservation of neurological function, several authors have reported the advantages of surgery combined with radiation, or surgery alone, compared with radiation alone (Klimo et al. 2005, Patchell et al. 2005). One prospective, randomized trial showed that decompressive surgery plus radiation is favorable for both preservation and regaining of ambulatory function compared to radiation alone, but even in these highly selected patients median survival was only 4.2 months (Patchell et al. 2005). Furthermore, when the same data were stratified according to age, the authors found that at ≥ 65 years the beneficial effect of surgery fades to become equivalent to that of radiation therapy alone (Chi et al. 2009).

Many patients with advanced prostate cancer are elderly, and have limited physiological reserve. The presence of visceral metastases will further depress their tolerance to surgery and render them susceptible to complications, thus having a potentially negative effect on survival (Bauer and Wedin 1995). In our study, patients with bad performance status and those with visceral metastasis had worse survival. Thus, 5 of 11 patients with KPS of ≤ 60 had visceral metastases and 7 of these 11 patients had complications after surgery. Furthermore, in our previous study high preoperative serum PSA, probably related to heavy tumor burden, was found to be associated with shorter survival in patients with hormone-refractory tumors (Crnalic et al. 2010).

In the present study, patients with hormone-naïve tumors had good functional outcome and two-thirds were still alive at the latest follow-up. Indeed, using the primary tumor diagnosis as a starting point in survival analysis, the expected postoperative survival for this patient group could be similar to the survival of the hormone-refractory group after primary diagnosis (Figure 1b). The effect of acute orchiectomy on reduction of spinal tumor volume and improvement of functional outcome in these patients should not be underestimated. However, in 4 of our patients neurological symptoms continued to progress despite acute orchiectomy, thus leading to spinal surgery after 2–7 days. There have been few reports on hormone-naïve prostate cancer with spinal cord compression. Huddart et al. 1997) reported on 17 patients. 13 were operated and 4 received radiotherapy, with a median survival of 1.7 years after treatment. These authors concluded that the absence of prior hormone therapy was a prognostic factor for better survival. In a study by Jansson and Bauer 2006), 13 patients with previously untreated prostate cancer had a survival rate of 62% two years after surgery for spinal cord compression, which is similar to our results. However, results of radiotherapy alone for spinal cord compression in 37 patients with hormone-naïve prostate cancer showed comparably poor median survival (11 months) and worse functional outcome (only 6 of 37 patients had improved motor function) than for the patients with known metastatic disease (Rades et al. 2006).

In summary, our findings indicate that patients with hormone-naïve disease and those with hormone-refractory disease with good performance status and no visceral metastases may be helped by surgery for metastatic spinal cord compression.
